# The Role of Micronutrients in Ageing Asia: What Can Be Implemented with the Existing Insights

**DOI:** 10.3390/nu13072222

**Published:** 2021-06-29

**Authors:** Taichi Inui, Bryan Hanley, E Siong Tee, Jun Nishihira, Kraisid Tontisirin, Peter Van Dael, Manfred Eggersdorfer

**Affiliations:** 1 DSM Nutritional Products, Tokyo 105-0011, Japan; 2 Academic Centre for Dentistry Amsterdam (ACTA), Department of Oral Microbial Ecology, 1081 LA Amsterdam, The Netherlands; a.b.hanley@acta.nl; 3 Nutrition Society of Malaysia, Petaling Jaya 46150, Selangor, Malaysia; president@nutriweb.org.my; 4 Department of Medical Management and Informatics, Hokkaido Information University, Hokkaido 069-8585, Japan; nishihira@do-johodai.ac.jp; 5 Institute of Nutrition, Mahidol University at Salaya, Nakhorn Pathom 73170, Thailand; kraisid.tontisirin@gmail.com; 6 DSM Nutritional Products, CH-4303 Kaiseraugst, Switzerland; peter.van-dael@dsm.com; 7 Department of Internal Medicine, University Medical Center Groningen, 9713 GZ Groningen, The Netherlands; manfred.eggersdorfer@dsm.com

**Keywords:** micronutrient, DALY, vitamins, LC-PUFA, carotenoid, nutritional status

## Abstract

Life expectancy as a measure of population health does not reflect years of healthy life. The average life expectancy in the Asia-Pacific region has more than doubled since 1900 and is now above 70 years. In the Asia-Pacific region, the proportion of aged people in the population is expected to double between 2017 and 2050. Increased life expectancy leads to an increase in non-communicable diseases, which consequently affects quality of life. Suboptimal nutritional status is a contributing factor to the prevalence and severity of non-communicable diseases, including cardiovascular, cognitive, musculoskeletal, immune, metabolic and ophthalmological functions. We have reviewed the published literature on nutrition and healthy ageing as it applies to the Asia-Pacific region, focusing on vitamins, minerals/trace elements and omega-3 fatty acids. Optimal nutritional status needs to start before a senior age is reached and before the consequences of the disease process are irreversible. Based on the nutritional status and health issues in the senior age in the region, micronutrients of particular importance are vitamins A, D, E, C, B-12, zinc and omega-3 fatty acids. The present paper substantiates the creation of micronutrient guidelines and proposes actions to support the achievement of optimal nutritional status as contribution to healthy ageing for Asia-Pacific populations.

## 1. Introduction

The global reduction in infectious disease has been a landmark achievement over the past century due to effective therapeutic and vaccine developments as well as medical practices. This situation remains fragile as shown by the HIV and SARS-CoV-2 virus outbreaks, particularly highlighted by the rapid and global impact of SARS-CoV-2, classified by the WHO as a pandemic. The death rate from SARS-CoV-2 in 2020 was approximately 1% of those infected, resulting in more than 2.5 million deaths globally to date [1]. In comparison, deaths due to non-communicable diseases (NCDs) are unfortunately significantly greater and get less media and public attention. In 2019, premature deaths due to cardiovascular disease were estimated to be 18.6 million and the global burden was 523 million cases [2].

NCDs have a significant negative impact on mortality and quality of life, which increases with age [3]. The risk presented by many NCDs is modifiable by lifestyle including diet. While several nutritional factors play a role in most NCDs inadequate micronutrient status is often associated with increased incidence and severity of NCDs. Older populations in particular suffer more from the additive effects of NCDs and have reduced resilience and ability to maintain healthy homeostasis [3]. Optimal micronutrient status throughout life increases resilience and supports more healthy homeostasis in the ageing population [4]. It remains a challenging question as to which micronutrients are most beneficial. This review assesses the association between specific micronutrients and health endpoints in ageing populations in the Asia-Pacific region, which hosts the largest ageing population. The assessment enables the recommendation of effective dietary interventions throughout life that will contribute to improve public health and reduce the negative effect of suboptimal micronutrient status. The review of the scientific evidence is based upon two criteria:

- The significance of the health issue, namely, which NCDs create the greater individual and societal burden.
- The strength of evidence between the nutrient status/intake and the incidence/severity of NCDs. The strength of evidence is graded as:
  ○ Associative, when evidence is based on associational data, but data from intervention studies are not yet available
  ○ Probable, when the evidence is based on both associational data and a plausible mechanism of action for the association
  ○ Convincing, when there is a high level of causality as demonstrated in intervention studies, supported by data regarding the mechanism of action.

Based upon this assessment recommendations are proposed for the development guidance to optimize micronutrient intake throughout life in support of healthy ageing. While the current assessment focuses primarily on the Asia-Pacific region, it may also apply globally as many trends are similar.

### 1.1. Ageing Populations

Asia hosts the most rapidly ageing population in the world. In 2017, 12% of the population in Asia was aged ≥ 60 years, a number that is projected to reach 24% by 2050 [5]. Japan, for example, is currently the only Asian nation that is defined as ‘superaged’, with more than 20% of its population ≥ 65 years. Other Asian nations, such as China, South Korea, Thailand and Singapore, are expected to reach this stage by 2030, resulting from a combined increase in life expectancy and a drop in birthrate. Among the Association of Southeast Asian Nations (ASEAN) countries, the average life expectancy is 71 years, reaching over 75 years in Thailand, Vietnam and Malaysia and just under 83 years in Singapore. Combined with declining birth rates the potential problem will likely be exacerbated as life expectancy continues to increase and the birth rate remains below that required for population replacement (Table 1). Therefore, the demographic will continue to shift towards an increasingly aged population.

**Figure 1:**
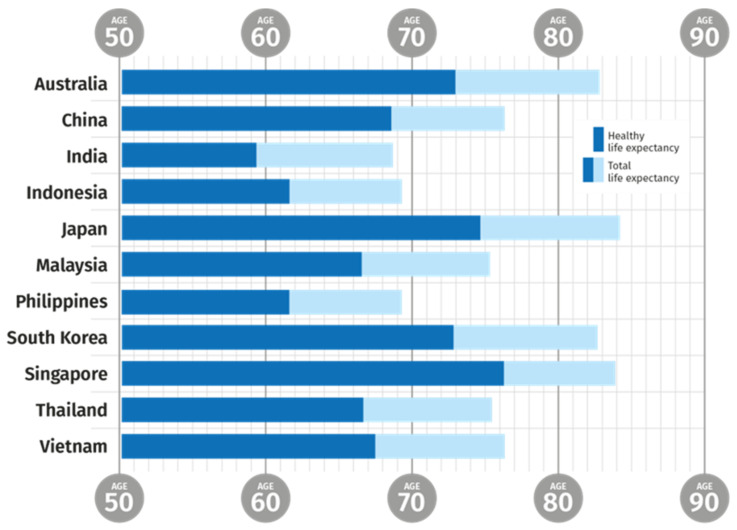
Gap between total life expectancy (TLE) and healthy life expectancy (HLE) among selected Asia-Pacific countries. TLE, Total Life Expectancy; HLE, Healthy Life Expectancy. Data for both sexes [7].

Consequently the potential support ratio available to take care of the retired generation decreases and increases the load per capita on the working generation [6]. Five countries in the Asia-Pacific region, (Australia, Japan, New Zealand, South Korea and Singapore) are expected to have a potential support ratio less than three by 2030. The pressure is increased by the gap between overall life expectancy and healthy life expectancy, which remains at 8 to 10 years (Figure 1) [7].

### 1.2. Age and Health

The increase in life expectancy has resulted in an increase in people with reduced healthy life expectancy [7]. Reduced health and wellness in the ageing population leaves a substantial number of people with a decreased quality of life (QoL) and increased dependence on healthcare support. The WHO reported that life expectancy globally increased from 66.8 years in 2000 to 73.4 years in 2019. The healthy life expectancy (HALE) on the other hand also increased from 58.3 in 2000 to 63.7 in 2019, which indicates that the increase in HALE (5.4 years) has not kept pace with the increase in life expectancy (6.6 years) [7]. In other words, people are living longer, but with reduced healthy life spans. This is further illustrated by figures for disability adjusted life years (DALYs)—a measure of the loss of health—one DALY represents the loss of one year of full health. DALYs due to communicable diseases have dropped by 50% since 2000, while those due to NCDs have increased [8].

There is an additional aspect to health and disease depending on economic circumstance. While about 23% of the total global burden of disease is related to disorders in people ≥60 years , the proportion of the burden is highest in high-income regions [4]. In addition, it has to be noted that countries with similar overall age-related burden, experience different onsets of ageing. For example, 76-year-olds in Japan and 46-year-olds in Papua New Guinea have the same level of age-related disease burden as the global average 65-year-olds, indicating that age alone is not a sufficient measure of disease burden [7].

Diet is considered a major factor associated with increased prevalence of chronic (non-communicable) diseases. The recent Global Burden of Disease study showed that in 2017, 11 million deaths and 255 million DALYs were attributable to dietary risk factors. High sodium intake and low intakes of whole grains and fruits were the leading dietary risk factors for death and DALYs both globally and in many individual countries [9]. This supports the impact of sub-optimal overall nutritional status on health, in terms of functional ability and independence as well as mortality. Nutritional status is associated with the occurrence of disease, and in particular with NCDs in older populations. The number of comorbidities, the occurrence of more than one disease condition at the same time, is one of the leading challenges in achieving healthy ageing in developed nations, and dietary risk is an important risk factor for comorbidity [9,10].

While several nutritional factors have been associated with disease prevalence, intervention studies clearly establishing a causal effect with a disease state are still relatively rare. For example, the higher intake of omega-3 long-chain polyunsaturated fatty acids (LC-PUFAs) was associated with health benefits in cognitive health, age- and disease-related decline in muscle mass during cancer treatment [11]. However, there are several causes of general malnutrition in the elderly that influence muscle mass, leading to sarcopenia and cachexia [12,13]. Malnutrition, in turn, can increase the susceptibility to and prevalence of chronic disease and make cause and effect difficult to define in the context of specific micronutrient dietary components [12]. Meeting this challenge requires the three components of association, probability and convincing causality to be identified in order that the extent of the contribution of micronutrient status to a given NCD will be able to be addressed. Even then, the solution is likely to be partial rather than definitive. However, that is the case for the health–disease axis whether you are dealing with overt illness and a mechanism-based pharmacological intervention or NCDs and a nutritional intervention.

Optimal nutritional status in the elderly (even just in the context of micronutrient status) is complex due to a range of factors including multi-morbidity, definition of clinical endpoints, multiple contributory confounding factors and increased susceptibility/decreased resilience to the impact of dietary factors on health [14]. There is a recognized need for “an effective, personalized, and scientifically based model for the assessment and evaluation of nutritional status in old people” [15]. This model should include micronutrient status and will vary depending on genetic (and epigenetic) predisposition and dietary and medical history [16].

Three of the top five methods to improve global welfare outlined in the 2008 Copenhagen Consensus are related to improving micronutrient status [17]. The recommendations acknowledge that optimizing nutrition throughout the lifetime is important in lowering the burden of increased chronic disease in older populations.

Nutrition-related disease incidence will increase in the Asia-Pacific region, and this will create a larger problem for those countries [6]. Measures to introduce science-based, evidence-led opportunities to sustain healthy ageing through nutrition are already underway. These have a clear focus on the role of micronutrients, including vitamins, minerals/trace elements, omega-3 LC-PUFAs and essential carotenoids. The active involvement of all stakeholders representing science, policy and industry can provide guidance on the role of nutrition in the maintenance of healthy ageing.

## 2. Obstacles to Healthy Ageing in Asia

**Figure 2:**
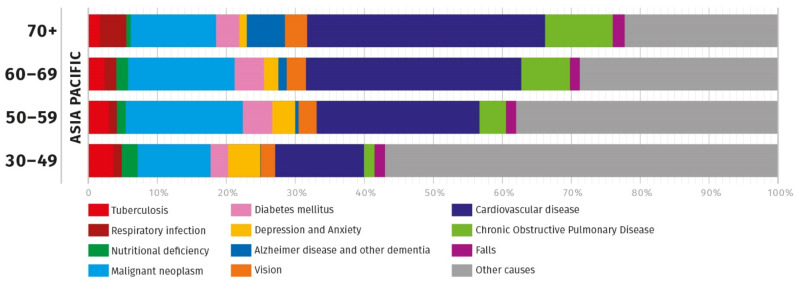
Top causes for DALY in the Asia-Pacific region per age group [3]. Asia-Pacific data were based on weighted arithmetic means by population between data from the Southeast Asia Region and the Western Pacific Region. Some causes were combined to group the relevant GHE codes—for example, “vision” is a sum of five visual organ disorders under ‘sensory organ disorders’ with GHE codes 1030–1070.

There are a number of obstacles to healthy ageing and these vary in severity and impact [4]. The WHO has defined the term ‘healthy ageing’ as “the process of developing and maintaining the functional ability that enables well-being in older age” [4]. The measurement of DALY for different conditions is a useful marker for the loss of functional ability and is, therefore, a measure of the diminishment of healthy ageing. Based on the DALY for different conditions in the Southeast Asian and the Western Pacific regions, the leading health issues among the middle age and elderly include eye health (sensory organ diseases), cardiovascular diseases (CVD), cognition (neurological disorders), reduced mobility, frailty (falls) and immunity (upper respiratory infection) (Figure 2) [3]. Individual countries report variations of the top issues within these leading causes.

The incidence rate of CVD in the Asia-Pacific region, in general is on the rise, driven primarily by stroke and ischemic heart disease (IHD). The highest stroke incidence in the region is in China, at 403 cases per 100,000 people, followed by Hong Kong and Japan [3]. The IHD incidence is highest in Japan and Hong Kong, with approximately 396 cases and 365 cases per 100,000 people, respectively, in 2019.

CVD is the cause of over 40% of deaths in the Chinese population [18]. It was the leading cause of death in China in 2019, accounting for 45.50% and 43.16% of all deaths in rural and urban areas, respectively. The absolute numbers and rates per 100,000 population for all-age DALYs increased substantially for stroke and ischemic heart disease between 1990 and 2017 (DALY counts increased by 46.8% and 125.3%, respectively). The risk factors that account for the greatest disease burden in China are, in priority order, dietary risks, high blood pressure and tobacco smoking.

In India in 2017, there were about 9.7 million deaths and 486 million DALYs so the ratio of DALYs to deaths was about 50 to 1 [19]. The ratio of DALYs to population in 2019 increased from 1.1 in the 45–49 years group to 2.1 in the 60–64 years group and further to 4.5 in the 85 years and older group [3]. In addition, the proportion of DALYs due to NCDs exceeded 50% in the 30–34 years group and was highest at 78.8% in the 65–69 years group.

Elsewhere in Asia, the incidence of NCDs has also been rising. In Malaysia, for example, the prevalence of diabetes was 17.5% in 2015 and is reported to have increased to 18.3% in 2019 [20,21]. In both reports, the prevalence was highest in the oldest age groups. In Japan, there have been increases in dementia, CVD (stroke), frailty and fracture [22]. Nutritional deficiency appears to account for a lower amount of DALY because the definition only includes classic deficiency symptoms.

One of the most significant shifts in recent years is the transition from the predominance of infectious diseases to NCDs [10]. Four-fifths of NCD-related deaths are in low- and middle-income countries, with older people in developing countries particularly at risk.

Dementia is a major NCD in elderly populations. There were 22.9 million cases reported in 2015 in Asia. This number is expected to rise to 38.5 million in 2030 and 67.2 million in 2050 [23]. Globally, the cost of dementia is over USD $817 billion and is expected to rise steeply. Of the 35 million people currently living with dementia globally, 58% live in low- and middle-income countries and by 2050 this figure is projected to reach 71% of the total. According to the WHO, Eastern Asia and Southern Asia will see dementia growth rates more than double in the next 20 years [24]. In Singapore in 2013, the cost of dementia per person was estimated at S$10,245 (USD $7700) per year (95% CI, S $6954 to S $12,495). Approximately 18.12 million people are projected to have dementia by 2030 in China. Extrapolating from the cost in Singapore, this could cost China an estimated USD $150 billion per year or about 15% of their annual healthcare budget.

The health-care systems of countries have been built and developed to provide acute medical care. The problem of preventing or managing chronic disease is exacerbated by the issue of comorbidity where diseases are rendered more impactful by their co-occurrence with other conditions that can affect recovery or cause further decline. Comorbidities of chronic diseases increases in likelihood with age. Globally, the prevalence of comorbidity is 21.3% in those aged 65 years and above, compared to 3.8% for those aged 18–49 years [25]. In the WHO’s Study on global AGEing and adult health (SAGE), the prevalence of comorbidity in the age groups 60–69 and 70 years and above was 50% and 60%, respectively [26]. Comorbidity not only affects physical functioning, emotional states and quality of life, but also increases the cost and complexity of care for these individuals and thus creates unanticipated challenges for health systems [27]. For example, a link between a chronic somatic disease (e.g., diabetes) and depression has been found among patients in eight Asian countries (China (including the Mainland, Hong Kong SAR, Taiwan), India, Indonesia, Japan, the Republic of Korea, Malaysia, Singapore and Thailand) and it has also been found that this comorbidity has clinical significance [28].

Immune function is affected by age. The age-related diminished responsiveness of the immune response has been termed immunosenescence, which impacts both the innate and the adaptive immune system [29]. Chronic, low-grade inflammation, which can in part be caused by dysregulation of innate immunity, increases with age, and this can also affect a number of NCDs including atherosclerosis, Alzheimer’s disease, osteoporosis and diabetes [29].

Visual impairment has significant effects on the quality of life and daily life, including mobility, such as walking and driving, and identifying people. People with vision loss are at higher risk for several types of injury and, in addition, those with vision impairment are at a higher risk for depression, anxiety and other psychological problems [30]. A study in the US in 2013 found that direct medical expenses, other direct expenses, loss of productivity and other indirect costs for visual disorders across all age groups were approximately USD $139 billion [31]. A systematic review and meta-analysis of blindness and distance vision impairment was published in 2017. A conclusion of the study was that “the number of people affected by the common causes of vision loss has increased substantially as the population increases and ages” [32]. According to a WHO Report [33] almost 70 million people in Asia suffer from age-related macular degeneration (AMD). The report also notes that “Globally, at least 2.2 billion people have a vision impairment, and of these, at least 1 billion people have a vision impairment that could have been prevented or is yet to be addressed”.

## 3. When Does the Burden of Ageing Commence?

Nutritional deficiency is defined as a clinical condition or disease linked to a specific nutritional deficit (Table 2). Instances of nutritional deficiency have been studied over many years and are responsible for global dietary recommendations and, where appropriate, fortification. The prevalence of actual nutritional deficiency as measured by the occurrence of nutritional deficiency diseases, decreases in the 50–59 age group; however, it increases again after age 60.

Developing a link between functional markers of health rather than disease and linking these to diet is more complex. It has been noted that older adults have increased requirements for specific macro and micronutrients, especially protein, calcium, vitamin D and B-vitamins in the context of the link between healthy eating patterns and healthy ageing [34]. This indicates the need for studies, information and action to maintain function in older populations in addition to overt nutrient deficiency disease prevention. The onset and extent of measurable changes due to NCDs, the deterioration of health conditions and the loss of quality of life associated with age will vary depending on the health indices being measured and the individual. There is no consensus as to when the initiation or progression of age-associated conditions takes place, although most population-based studies set 65 years old as the start point for ageing, following the classification of UN population studies. However, a large number of conditions are already able to be assessed before that age. Since some NCDs haver a long gestational period, earlier interventions may slow their onset and a number of these interventions may be nutritional in nature.

Biological ageing can be defined in terms of three key variables:

- programmed ageing of specific components (e.g., cells, organs);
- ageing caused by cumulative damage and a loss of resilience to that damage (e.g., cumulative damage to DNA or to other biological macromolecules); and
- general wearing out of body parts and functions due to extended use (loss of fitness and resilience).

One of the most significant aspects of *programmed ageing* is telomere length. The ends of human chromosomes are protected by DNA–protein complexes termed telomeres. The telomeric DNA shortens with repeated cell divisions until the telomeres reach a critical length, at which point the cells enter senescence. Telomere length has been proposed to be a biomarker of cell age and is highly correlated to chronological age and metabolic status. Diet has been found to influence the maintenance of telomere length [35]. Telomeres may, therefore, be a useful investigative tool to measure the effects of diet and specific nutrients or classes of nutrients on programmed cell ageing and on age-related disease. Indeed, accelerated telomere shortening has been found in Asian male populations with Type 2 diabetes so using nutrition to lessen the impact of metabolic disease on ageing through prevention of telomere shortening may be possible [36]. One of the most significant aspects of *cumulative damage* to biological macromolecules and subsequent ageing is that brought about by a range of mechanisms [37]. As these authors noted “Because no single type of damage, or cellular response to damage, is likely to act alone, a systems approach will be needed to connect damage type, underlying cause, and tissue-specific aging phenotypes. These connections will also be necessary to design and assess strategies for mitigating damage to postpone aging phenotypes”. The same argument applies to dietary components that restrict ageing. Not all of them will act in the same way or upon the same mechanism of cumulative damage leading to ageing [38].

Resilience to the ageing processes is also termed *fitness*. Optimal nutrition can help to build resilience to the effects of ageing and increase fitness [4]. In some cases, ageing is a linear progression while in others there is an inflexion point where accelerated decline occurs. Whether linear or accelerated, the process will gradually reduce the fitness of the biological system such that it will eventually no longer be able to function effectively. In order for a nutritional (or, indeed, any) intervention to be effective, it must occur at a point in the ageing process where it will either halt or reverse the consequences of the biological ageing process. This is harder to accomplish if the system is in accelerated decline. Halting or reversing the ageing process or preventing the progression to accelerated decline can reduce the numbers of DALYs and improve the quality of life. While the significance of NCDs increases with increased age, a number of these are also major causes of DALYs at earlier ages, so there may be a doubled advantage to improving health and fitness in the immediate term and slowing of the loss of fitness as one gets older. The proportion of DALYs attributed to a range of disease states is shown in Figure 2. The main causes in terms of relative impact are CVD and Alzheimer’s Disease. Between them, these account for about 40% of DALYs for those over 70 years of age and 15% in those aged 30–49 years in the Asia-Pacific Region. The extent to which earlier interventions may ameliorate later incidence of disease giving rise to DALYs has been comparatively little studied. However, it is known that certain classes of nutrients may delay programmed ageing, help to diminish the impact of cumulative damage and improve resilience as described in the following section.

Any discussion of when the burden of ageing begins has to take into account the rate of accretion of NCDs among different age groups. There is an increased prevalence of NCDs at ages 50–59 compared to ages 30–49 [3]. As the development of most NCDs is a gradual process, this suggests that there may be windows of opportunity for interventions that lead to risk reductions of diseases developing later in life. The focus for such interventions should be on NCDs before age 50. An early beginning of interventions may be of particular preventive value and may divert the disease such that progress towards a deleterious clinical outcome may be slowed, delayed or prevented. An example of this is bone mass, which peaks in the 20s and 30s and gradually declines afterwards. Suboptimal lifestyle factors when loss in bone mass is being accreted (including nutritional status) can create a higher risk of osteoporosis, fracture risk and other skeletal issues at later life stages [39]. Examples of early onset of disease have been observed and studies of adults from a birth cohort (n = 954) indicated a decline in the integrity of multiple organ systems associated with biological ageing and associated biomarkers as early as 38 years old [40]. In many cases, while the actual burden is low and may only be detected clinically or signified by predictive biomarkers, interventions may be more effective at an early stage. Although this may be a preferred approach, it is often challenging to identify the underling health issue that is yet to develop to the diagnosable stage because the transition between being healthy and being ill is continuous, and often no explicit borderline exists. Measures based on minor physical and mental complaints (MPMC), such as stress, sleeplessness, irritation, lethargy and loss of appetite has been proposed to fill this gap (personal communication, Yamamoto-Maeda). A panel of biomarkers used in MPMC includes a questionnaire of dietary habits, haematological examination, endocrine function, immune function, sleep assessment, physical and mental stress and population of gut microbiota. It is noteworthy that MPMC focuses on the stage closer to being healthy in the overall spectrum between healthy and unhealthy. Therefore, the complaints and symptoms in this stage can often be reversed towards health through personal behaviours, including the intake of healthy food and nutrients. Most of the complaints and symptoms observed in MPMC are frequently seen as a consequence of ageing.

## 4. Role of Nutrition in Healthy Ageing in Asia

Nutrition is a mediator between food and health that acts by supporting health and wellness as a result of long low-dose (lifetime) exposure. Many NCDs begin in healthy middle age but are only clinically manifest in later life, by which time reversal by modification of diet cannot be carried out and medical or pharmaceutical intervention is the only option. Changes in diet, metabolism, physiology, hormone balance, exercise and exposure to a range of bioactives can affect the onset of NCDs which, in turn, will further affect optimal nutrition requirements. The most significant of these will be considered in this review.

Optimal nutrition can promote healthy ageing prior to nutritionally irreversible disease being diagnosed. Nutritional status, therefore, is a measure of the overall level of nourishment and is an important factor in assessing the quality of life, morbidity and mortality [41]. This leads to the key principles that underlie the role of nutrition in healthy ageing—first of all, there must be a scientifically demonstrable link between a specific dietary component (or set of components) and a clinically provable NCD. Secondly the relationship must be such that a dietary modification, at an appropriate timepoint, must lead to a reduction in the prevalence or risk associated with the NCD. While there should be some causality, it is important to distinguish between pharmaceutical outcomes targeted towards specific disease endpoints and nutrition but also between overt nutrition deficiency diseases and NCDs.

The following sections characterise and summarise the key health issues associated with ageing and the potential role of micronutrient interventions from recent human studies. The examples have been chosen based upon the strength of the evidence base and the likely success of an intervention.

### 4.1. Cognition

Nutritional factors—B vitamins including folate, omega-3 LC-PUFAs and carotenoids (lutein)

Evidence—B vitamins including folate—Probable/Convincing: omega-3 LC-PUFAs—Probable: carotenoids (lutein)—Associative/Probable

The roles of B vitamins and omega-3 LC-PUFAs (DHA) in maintaining healthy psychological as well as brain functions are acknowledged by authoritative bodies such as the EFSA [42].

Cognitive decline in people at older age is a major issue. The brain begins to shrink at a rate of 0.5% per year in persons over the age of 60 [43]. People with mild cognitive impairment show a more rapid shrinkage rate of ca. 1.0% per year, and in patients with Alzheimer’s disease, the rate was even higher, at 3% per year. From a nutritional standpoint, atrophy in the cerebral cortex and in the hippocampus has been associated with elevated homocysteine levels. Homocysteine is converted into methionine with using the nutritional cofactors, vitamin B-6, folate and vitamin B-12, thus reducing its concentration. An elevated plasma homocysteine level is a biomarker for B vitamin status [44]. A recent International Consensus Statement concluded that elevated plasma total homocysteine is a modifiable risk factor for development of cognitive decline, dementia, and Alzheimer’s disease in older persons [45]. The study also concluded that “the public health significance of raised tHcy (serum total homocysteine) in the elderly should not be underestimated, since it is easy, inexpensive, and safe to treat with B vitamins”. Atrophy rates were reduced in individuals taking dietary supplements containing vitamin B-6, folic acid and vitamin B-12 and who had sufficient omega-3 LC-PUFAs status [43,46].

A population-based prospective cohort study tracked 16,948 subjects for the dietary intake of six B vitamins in midlife with cognitive impairment in old age in Singapore over 20 years. The results indicated that riboflavin (vitamin B-2) and folate intake were inversely associated with cognitive impairment in a dose-dependent manner, whereas dietary intake of thiamine, niacin, vitamin B-6 and B-12 were not significantly associated [47]. The intake of docosahexaenoic acid (DHA) alone or in combination with eicosapentaenoic acid (EPA) has been shown to support memory health in older adults with mild memory complaints [48]. A review of randomized clinical trials (RCT) indicated that individuals with low dietary intake of omega-3 LC-PUFAs and with age-related cognitive decline or mild cognitive impairment have been suggested to benefit the most from the consumption of additional omega-3 LC-PUFAs [49].

One underlying mechanism suggested a role of micronutrients with their antioxidant properties. Emerging science has shown the role of antioxidant carotenoids in cognitive health. Post mortem concentrations of brain lutein, but not alpha-tocopherol, in the subset of centenarians without dementia were significantly and positively associated with a range of cognitive measures [50]. Studies on the effects of whole diets on cognition have continued to be carried out. The potential role of an adapted Mediterranean diet as a modifiable dietary factor to reduce the rate of cognitive decline in later life in a Chinese population has been demonstrated [51].

### 4.2. Mobility (Musculoskeletal Health)

Nutritional factors—vitamin D, calcium, other minerals (bone and muscle mass), protein (muscle mass), omega-3 LC-PUFA (muscle mass)

Evidence—vitamin D + calcium—Probable/Convincing: other minerals—Associative: protein (bone)—Associative: protein (muscle)—Probable: omega-3 LC-PUFA (muscle)—Associative/Probable: vitamin D (muscle)—Convincing.

The roles of vitamin D and calcium, as well as other minerals such as magnesium, manganese, phosphorus and zinc, in maintaining healthy bone and muscle functions are acknowledged by authoritative bodies such as the EFSA [42,52].

The risk of fractures increases by a factor of 10 every 20 years, due to increased risk of falling as well as changes in the bone architecture. Osteoporotic fractures count for more hospital days than many other diseases, including diabetes, myocardial infarction and breast cancer [53]. While bone mass is built up during early life (up to about 30 years of age) in the period between 30 and 50 years of age there is a balance between bone resorption and accretion. There are a number of factors that influence bone health including protein intake, vitamin D status and calcium intake as well as lesser-known components that have been suggested such as magnesium, silicon, vitamin K and boron.

Bone contains considerable amounts of protein, and an adequate intake of protein is essential in building peak bone mass and in the maintenance of bone during middle aged adulthood. It was suggested previously that a diet rich in compounds whose metabolism generates acid (e.g., proteins) would lead to low-grade metabolic acidosis, thereby impairing osteoblast function, stimulating osteoclast survival and activity, increasing bone resorption, and decreasing bone mass and strength. However, a recent consensus review found that, in an assessment of systematic reviews and meta-analyses addressing the benefits and risks of dietary protein intakes for bone health in adults, dietary protein levels above the current RDA may be beneficial in reducing bone loss and hip fracture risk, provided calcium intakes are adequate. The review concluded that there is no adverse effect of higher protein intakes on bone health, there may even be benefits in attenuating age-related bone loss and reducing hip fracture risk and that a causal link between dietary acid load and osteoporosis is not supported by clinical evidence [54]. Most recent studies conclude that, if the body is in mineral balance, higher intakes of dietary protein do not have any detrimental effect on bone and likely offer a beneficial effect [55].

Micronutrients are also important for bone health, in particular vitamin D and calcium intakes are recognized as significant contributors to bone health [39]. Dietary sources of vitamin D are limited to a few food groups. In addition, both calcium and phosphorous are essential for correct bone mineralization. In the case of vitamin D deficiency, there is a 15% drop in the absorption of calcium and up to 60% in that of phosphorus [56]. Several studies in humans have shown that bone mineral density increases with higher 25(OH)D plasma concentration. A plasma concentration of 25(OH)D above 75 nmol/L is considered optimal and may be achieved by the daily intake of 20–25 µg (800–1000 IU) vitamin D [57]. In a meta-analysis, it was shown that this vitamin D intake can be expected to reduce bone fractures by up to 20%. The potential savings in healthcare costs due to a reduced number of bone fractures by the administration of vitamin D and calcium supplements is estimated at up to EUR 3.96 billion per year in Europe [58]. The relationship between bone mineral density and vitamin D status in Asian populations remains somewhat conflicted. One review [59] suggested that, based on an analysis of published data, “the evidence for a positive relationship between the serum 25(OH)D concentration and bone mineral density (BMD) in the middle-aged and older Chinese population living in Asia appears to be limited and inconsistent”, however, the authors acknowledge that “few studies were available and most of the 11 selected studies were cross-sectional in design and susceptible to bias. Moreover, most of the studies were not specifically conducted to examine a relationship between serum 25(OH)D concentrations and BMD, and not all studies used the same cut-off point to define vitamin D insufficiency and deficiency”. The WHO have stated that “For older people, there is convincing evidence for a reduction in risk for osteoporosis with sufficient intake of vitamin D and calcium together” [60].

Maintaining muscle strength is another key factor for mobility. In addition to proteins, micronutrients including calcium, magnesium, potassium, vitamin D and omega-3 LC-PUFAs have been known to play a role in maintaining muscle functions. Daily supplementation of 2 g fish oil accompanied by strength training improved muscle strength and functioning in elderly women compared to a group receiving strength training only [61,62].

A systematic review and meta-analysis of RCTs (adults > 60 years of age) on the effect of vitamin D without additional exercise on muscle function revealed a consistently beneficial effect on muscle strength and balance for daily vitamin D doses of 20 to 25 µg (800–1000 IU) [63]. Another systematic review of the elderly reported a beneficial effect on muscle strength, but not muscle mass and muscle power, for supplemental vitamin D intake with or without additional calcium supplementation [64]. The effect was more significant for participants with low 25(OH)D concentration (<30 nmol/L) and for older participants (≥65 years). In addition, a cross-sectional study based on data from the South Korea National Health and Nutrition Examination Survey with postmenopausal Korean women showed that higher 25(OH)D concentration was associated with lower risk of sarcopenia [65].

### 4.3. Diabetes

Nutritional Factors—Micronutrients (glucose metabolism), Vitamin C, Vitamin E (cardiovascular events)

Evidence—Micronutrients—Associative/Probable: Vitamin C—Probable: Vitamin E specific genotype—Convincing.

Age is a risk factor for type 2 diabetes, along with unhealthy dietary habits, sedentary lifestyle, high BMI and smoking [66]. A diabetes prevention diet aims to help weight loss and the maintenance of lean body weight through the consumption of fewer calories and lower levels of fat and high-glycemic carbohydrates, while increasing the consumption of dietary fiber and fruits and vegetable. Multiple micronutrients are involved in glucose metabolism (e.g., thiamin and chromium), insulin metabolism (vitamin D, biotin, magnesium and zinc) and mitigation of oxidative stress (vitamin D, vitamin E and vitamin C) [67,68]. In a prospective study that tracked 21,831 individuals over the course of 12 years, there was an inverse association between plasma vitamin C levels and risk of diabetes [69]. A study designed to directly look at the effect of vitamin C suggested that, in a randomized cross-over study involving 4 months of supplementation with oral vitamin C (2 × 500 mg/d), individuals with type 2 diabetes experienced improved postprandial and 24-h glycaemia and decreased blood pressure after 4 months of vitamin C supplementation as compared to placebo [70].

There is an increasing accumulation of evidence linking micronutrient deficiency to diabetes both as a causative factor and as a consequence of the disease [71]. A systematic review and meta-analysis of the effects of individual micronutrients on blood pressure in patients with type 2 diabetes suggested that vitamin D and possibly vitamin C have beneficial effects on blood pressure, specifically in patients with type 2 diabetes [72]. Cardiovascular complications are a major comorbidity factor in diabetic patients. Clinical evidence suggests that 65% of diabetic patients will suffer a heart attack and a significant portion will die as a result of cardiovascular complications. Those with diabetes and the haptoglobin Hp2-2 genotype have a higher risk of mortality due to cardiovascular events, including atherosclerosis. Vitamin E supplementation has been shown to reduce and normalize the risk for cardiovascular events among these populations by preventing the oxidation of high-density lipoprotein [73]. The prevalence of the Hp2-2 genotype is higher among Asian populations compared to Caucasians. While 36% of Caucasians have this genotype, the prevalence is 72% for Indian ethnicities, 68% for Malays, 52% for Japanese and 45% for Chinese [74,75]. A clinically applicable prediction algorithm for long-term cardiovascular mortality in those with diabetes has been designed. Seven variables, including levels of 25(OH)D were selected for the final multivariate model [76].

### 4.4. Heart Function

Nutritional Factors—Omega 3 LC-PUFAs, vitamin D, vitamin C.

Evidence—omega-3 LC-PUFAs—Probable/Convincing: vitamin D—Associative: vitamin C—Associative. Possible effect on modifiable indices including blood pressure and lipid profile.

The roles of omega-3 LC-PUFAs (EPA and DHA) in maintaining healthy cardiac functions are acknowledged by authoritative bodies such as the EFSA [42].

Coronary heart disease and stroke are the two most common causes of mortality and morbidity for people over 60 years of age and are responsible for 17.3 million deaths per year accounting for approximately 31% of deaths globally [77]. The change in cardiovascular health is a lifelong process. Atherosclerosis develops over many years, and the risk factors, such as high blood pressure and increased cholesterol and triglyceride levels, increase with age in both men and women [78]. In addition to the impact of sodium reduction, two major human studies in the US have investigated the influence of nutrients, specifically vitamin D and omega-3 LC-PUFAs. In the *VIT*amin D and Omeg*A*-3 Tria*L* (VITAL) in the US, 2000 IU vitamin D and 2000 mg omega-3 LC-PUFAs were given to 20,000 adults over 50 years of age for the primary prevention of cancer and heart disease. The study reported a statistically significant reduction in coronary heart disease in the omega-3 arm (17% risk reduction) [79]. Although the effects of vitamin D were less pronounced, the evaluation showed that the study participants already had a vitamin D status in the optimal range at the beginning of the study, which suggested the impact of vitamin D supplementation might have been negligible. Overall, the results of this trial indicate that supplementation with either omega-3 LC-PUFAs at a dose of 1 g/day or vitamin D3 at a dose of 2000 IU/day was not effective for the primary prevention of CVD or cancer events among healthy middle-aged men and women over 5 years of follow-up. Another study, called REDUCE-IT, was conducted with an intervention in 8179 individuals with increased risk of CVD and taking statins. The intervention with 4 g omega-3 LC-PUFAs, in a form of EPA ethyl ester, compared to placebo resulted in a 25% risk reduction in the primary endpoints of cardiovascular death, myocardial infarction and stroke [80]. The results were further supported by health effects in the secondary endpoints including blood pressure and cholesterol levels. It should be noted that this study was a pharmaceutical study for secondary prevention, because it was conducted in a group of people who already had cardiovascular disease. High cholesterol and high blood pressure were mentioned as being among the top four modifiable risk factors that contribute to CVDs and are relevant across the Asian countries of China, Australia, Hong Kong, Japan, Singapore, South Korea, Taiwan and Thailand [81]. Micronutrients including DHA, EPA and vitamin C have been suggested to contribute to maintaining blood lipid profile and blood pressure in multiple meta-analyses [82,83,84].

### 4.5. Immunity

Nutritional Factors—vitamin D, vitamin C, zinc, omega-3 LC-PUFAs, other micronutrients (vitamins A, B-6, B-12 and E, folate, iron, selenium, magnesium and copper),

Evidence—vitamin D—Convincing: vitamin C—Convincing (during illness): zinc—Convincing (reduction of infection): omega-3 LC-PUFAs—Probable (effect on low grade inflammation), other micronutrients—Probable

The roles of vitamin D, vitamin C, folate, zinc in maintaining healthy immune functions are acknowledged by authoritative bodies such as the EFSA [42].

Immune functions decline with age, including the increased susceptibility to infectious diseases and poorer responses to vaccination [29]. In addition, immune function dysregulation also leads to a higher susceptibility to chronic inflammation, and this exacerbates a number of disease states. Several vitamins, including vitamins A, B-6, B-12, C, D and E and folate, along with trace elements including zinc, iron, selenium, magnesium and copper, play important roles in supporting the cells and tissues of the immune system [85]. Of those, the best available evidence is for vitamins C and D and zinc [86]. Deficiencies in or low levels of micronutrients have the potential to affect immune function negatively and may therefore decrease resistance to infections. An enhanced quality and an increased quantity in intake of antioxidants and other micronutrients is necessary to overcome the reduction in endogenous availability that occurs with ageing [87].

Because of the importance of the immune system in response to infection, much of the information regarding immune function relates to communicable diseases (CDs). A meta-analysis of individual participant data for nearly 11,000 people reported that, overall, taking vitamin D supplements reduced the risk of having at least one acute respiratory tract infection by 1242% [88,89,90]. The study found the greatest protective effects for those individuals with the lowest serum 25(OH)D concentrations (<25 nmol/L) taking a daily or weekly vitamin D supplement vs. a large dose taken at greater intervals. The risk of having at least one acute respiratory tract infection dropped from 60% to 28%. Other nutrients such as omega-3 LC-PUFAs also supported an effective immune system through modulating the host’s inflammatory responses [91].

Certain micronutrient requirements appear to increase during illness, such as vitamin C, the cytoplasmic concentrations of which decrease in the body during times of infection [92]. Supplementation of 45 mg elemental zinc/d for a year in elderly subjects (aged 55–87 years), has also demonstrated a dramatic reduction in the incidence of infection as well as plasma oxidative stress markers [93].

Tuberculosis remains the leading cause of death among bacterial infections, with the highest incidences occurring in Southeast Asia and Africa [94]. It has been suggested that tuberculosis is linked to low serum vitamin D concentration, which leads to attenuated antimicrobial responses to pathogens in macrophages [95]. A recent meta-analysis of individual participant data for RCTs showed that vitamin D supplementation accelerated sputum culture conversion, a positive prognostic marker for recovery from tuberculosis, in patients with multidrug-resistant pulmonary tuberculosis [96].

### 4.6. Eye Health

Nutritional Factors—omega-3 LC-PUFAs, Multiple supplementation (vitamins A, C and E, zinc, iron), Lutein, Zeaxanthin

Evidence—omega-3 LC-PUFAs—Convincing (in combination with other dietary components): Multiple supplementation—Convincing: Lutein/Zeaxanthin—Convincing.

The roles of omega-3 LC-PUFAs (DHA), vitamin A and zinc in maintaining normal vision are acknowledged by authoritative bodies such as the EFSA [42].

The prevalence of visual impairment increases sharply from the age of 60. The main triggers for impaired vision in the elderly population are amyotrophy, cataracts and AMD [97]. Oxidative stress is one of the main causes of lens opacification. Thus, antioxidant nutrients have been investigated for their roles in mitigating the risk of development of cataracts. One review that included observational studies found that vitamin C, beta carotene, lutein and zeaxanthin exerted the protective effect of antioxidation on cataracts [98]. A meta-analysis based on cohort studies and RCT concluded that supplementation of vitamin A, vitamin E, vitamin C, beta carotene, lutein and zeaxanthin was associated with the reduced risk of age-related cataracts [99].

In two large studies in humans (AREDS 1 and 2), it was demonstrated that the ingestion of a dietary supplement containing vitamin C, vitamin E, zinc, iron and the carotenoids lutein and zeaxanthin reduces the risk of the progression of advanced AMD [100]. Individuals taking lutein and zeaxanthin, alone or in combination with DHA and EPA, have a 10% reduction in the progression of advanced AMD. In addition, individuals with the lowest dietary intake of lutein and zeaxanthin showed a 26% reduction in the risk of progression to AMD. It is estimated that if the 8 million individuals in the United States who are at high risk of developing advanced AMD received the AREDS formulation, more than 300,000 of the 1 million persons expected to develop advanced AMD would avoid it and its associated vision loss [101]. A recent assessment for the European Union showed that a total potential saving of 6.20 billion Euros in medical costs per year could be realized if all persons with AMD risk took lutein and zeaxanthin supplements [102].

Although not included in DALY, dry eye is a health condition with particular relevance among the ageing population in Asia. While an average of 8% of the US population is affected, 27.5% in Indonesia, 30.3% in South Korea, 33% in Japan and 50% in China have at least one dry eye symptom with the prevalence increases for older adults [103,104]. A recent meta-analysis showed that omega-3 LC-PUFA supplementation improved dry eye symptoms and signs in patients with dry eye disease [105].

### 4.7. Nutrition in a Clinical Setting

While the role of nutrition in the ageing population in Asia is the primary subject of this review, it is acknowledged that nutrition also has a key role to play in the recovery of the elderly from clinical conditions requiring hospitalization or other interventions. This is more usually of a general nutritional type, however, there are also some specific nutrient imbalances that affect recuperation after illness.

Muscle atrophy and wasting is common among elderly hospital patients, especially among those who are immobilized and have pronounced inflammation. Hospital malnutrition is a prevalent and undertreated condition in stable patients, including patients with cancer and chronic obstructive pulmonary disease (COPD). The European Society for Clinical Nutrition and Metabolism (ESPEN) have published guidelines for the nutritional care of patients with cancer. These recommendations came against a background where it was estimated that the deaths of 10–20% of cancer patients were a result of malnutrition [106]. Malnutrition is also associated with longer hospital stays and increased healthcare costs [107]. Cancer-associated malnutrition promotes cachexia, a wasting syndrome characterized by weight loss. Elevated intake of protein as well as supplementation of branched-chain amino acids has been suggested to promote muscle protein anabolism and improve fat-free mass [108,109]. In addition, omega-3 LC-PUFA, especially EPA, has been shown to downregulate the inflammatory process and thus is suggested to contribute to the maintenance of muscle mass [107,110]. Both EPA and DHA have been shown to be beneficial and increasing the omega-3 to omega-6 LC-PUFA ratio in the diet was effective in decreasing tumor growth and cachexia development [111]. Similarly, a high-energy, high-protein diet is essential to correct malnutrition for stable COPD patients. A Cochrane systematic review evaluated the impact of nutritional supplementation in RCT on stable COPD patients and found a beneficial effect of nutritional intervention in the areas of weight gain and fat-free mass index, especially for malnourished patients [112].

## 5. Nutritional Adequacy and Nutritional Status in Asia

There are a number of terms that are used to describe different states of nutritional adequacy. Some of these are summarized as follows:

- Recommended Dietary Allowance (RDA): the average daily dietary intake level that is sufficient to meet the nutrient requirement of nearly all (97 to 98 percent) healthy individuals in a group.
- Adequate Intake (AI): a value based on observed or experimentally determined approximations of nutrient intake by a group (or groups) of healthy people—used when an RDA cannot be determined.
- Tolerable Upper Intake Level (UL): the highest level of daily nutrient intake that is likely to pose no risk of adverse health effects to almost all individuals in the general population. As intake increases above the UL, the risk of adverse effects increases.
- Estimated Average Requirement (EAR): a nutrient intake value that is estimated to meet the requirement of half the healthy individuals in a group.

These and other terms are based upon nutritional adequacy—not optimal intake. This in turn pivots around the definition of healthy. The implication (and practical result) of such reference values is that intakes that are lower than those recommendations will result directly in deficiency diseases. Optimal nutrition is most commonly used in relation to sports performance where the individual is seeking a performance level above that of the normal population and requires specific nutrition to help to achieve that, rather than healthy ageing. In addition, many nutrition studies, even where they are focused on older populations, tend to study the aged populations themselves rather than developing preventative strategies to be implemented at an earlier life stage.

A significant proportion of the adults and elderly population whose micronutrient intake level was below the Estimated Adequate Requirements (EAR) was reported in China, Japan, Philippines and South Asia, with older males being identified as a more vulnerable group for nutritional inadequacy [113,114,115,116]. A meta-analysis from 37 studies showed that, depending on the specific micronutrient, between 15% and 90% of the elderly were at risk of deficiency [117]. The prevalence of malnutrition in the elderly has increased substantially in the last 10 years in high-income countries [118]. In Asia, a significant proportion of the population is at risk for inadequate and deficient status for vitamin D [60,119], vitamin E [120], vitamin B-12 [121], folate [122], vitamin C [123], calcium [122], iron [122] and omega-3 LC-PUFAs [124]. Deficiencies in vitamin A and zinc remain to be an issue in Asia [125].

Global intake data suggest that populations with high blood levels of omega-3 LC-PUFAs were found in countries in East Asia (Japan, South Korea, and Primorsrky Krai region of Russia) while moderate blood levels were observed in Hong Kong, Mongolia and French Polynesia. Low levels were found in parts of the Asia-Pacific region (China, Russia, Singapore, Australia and New Zealand). Very low blood levels were observed in the Southeast Asia and India [124].

Low vitamin C status has been found throughout Asia in a review on global vitamin C status [123]. There is a shortage of well-defined epidemiological studies however in high income countries, a number of studies have suggested that the prevalence of deficiency is low but is measurable. Increased prevalence of low vitamin C status has been noted in the relatively few studies carried out in low- and middle-income countries.

The optimal nutritional status of individuals varies at different lifetime stages and hence deficiency is also variable according to age and physiological status. Low B-12 status as measured by blood levels (<148 pmol/L) and marginal deficiency (148–221 pmol/L) varies globally but is particularly prevalent in the South America, Africa and Asia with some countries exceeding 40% prevalence in different subpopulations [121]. Vitamin B-12 is important in folate metabolism and in particular the vitamin B-12-dependent conversion of homocysteine and 5-methyltetrahydrofolate to methionine and tetrahydrofolate. Therefore, vitamin B-12 deficiency is clinically linked to symptoms of folate deficiency and to high levels of homocysteine, which has been associated with cognitive decline in the elderly [44,45].

Much of the interest (and hence the studies) on folate levels has concentrated on women of childbearing age because of the link to neural tube defects and megaloblastic anemia. Older adults can be at risk as a result both of folate deficiency and overnutrition. High concentrations of unmetabolized folate (>45 nmol/L) have led to concerns about deleterious effects in older adults—especially when combined with low vitamin B-12 status [126]. A recent study (Irish Longitudinal Study on Ageing (TILDA)) shows that the prevalence of deficient or low B-12 status (<185 pmol/L) was 12%, whereas the prevalence of deficient/low folate status was 15%. High folate status (>45 nmol/L) was observed in 8.9% of the study population, whereas high B-12 status was observed in 3.1% (>601 pmol/L). High levels of unmetabolized folic acid has been suggested to be associated with some health issues [127] and a 2-year intervention suggested that higher folate levels were associated with lower total brain volume [44]. There have also been some studies suggestive of folic acid being associated with the progression of certain types of cancer, however these concerns have not been substantiated. Lower folate status has also been associated with a higher risk of all-cause mortality [128]. The consequences of vitamin B-12 and folate status in the general population in Asia and elsewhere remains unclear.

Many dietary studies in the elderly focused on a few specific vitamins, namely, vitamins B-6, B-12 and D, but overlooked the fact that a low dietary intake of one micronutrient can be accompanied by low intakes of other micronutrients present within that “inadequate” diet. Focusing on only one vitamin or mineral overlooks the fact that a micronutrient never occurs alone in the diet. Consequently, supplementation of one micronutrient alone is meaningful only if there is a clear indication, such as an isolated deficiency disease or some other physiological consequence of a sub-optimal intake [129]. Currently accurate intake as well as status data is hardly available. There are missing micronutrient intake data in the national dietary intake surveys in the majority of the Asian countries (Table 3). There is, therefore, a need for further studies to establish the micronutrient baseline for Asian countries and its consequences both on an individual and population level.

## 6. Factors Affecting Adequate Nutritional Status in the Elderly

There are multiple etiologies for micronutrient malnutrition, also known as ‘hidden hunger’, in the elderly. Changes in metabolism is one of the factors. Energy metabolism and lean body mass decrease with age, which leads to a decreasing energy need as well as lower intake. However, despite this reduced energy need, the need for micronutrients remains significant and has to be met. This creates an increased risk of hidden hunger among the elderly and is particularly relevant for those micronutrients which metabolism and absorption are impaired with age, such as vitamins B-12 and D [118].

Previously vitamin B-12 deficiency was recognized in higher income countries through patients having symptoms of pernicious anemia however this is caused by malabsorption (lack of production of intrinsic factor) rather than low intake. However, in low income countries, B-12 deficiency is more often a result of a low intake of B-12 rich foods—generally of animal origin [130]. More recently, it has been recognized that there is a high prevalence of sub-clinical deficiency—particularly in lower income groups. The rates of sub-clinical deficiency of vitamin B-12 are high in developing countries, in the elderly and in vegetarian populations. As with some other nutrients, vitamin B-12 requirements vary according to age and B-12 status varies during an individual’s lifetime. Deficiency is more common among older populations with 10-15% of people being found to have sub-clinical deficiency which can often be ameliorated by increased intake [131]. Prevention of B-12 deficiency in the majority of the population is best achieved by the consumption of B-12-rich foods however these are mostly of animal origin including dairy and eggs, and thus, there is a higher prevalence of vitamin B-12 deficiency among those for whom a high intake of animal products is not possible on economic, cultural or religious grounds. There have been numerous studies of vitamin B-12 status of Asian sub-populations in non-Asian countries who adhere to dietary habits from their ethnic background particularly vegetarian/vegan and a high proportion of the elderly in these populations are vitamin B-12 deficient [132]. The combination of ageing and consumption of vitamin B-12 depleted diets will exacerbate the clinical consequences of deficiency.

Another factor driving malnutrition among the elderly is polypharmacy which has been defined as the use of 5 or more oral prescription medications per month [133]. The proportion of older people taking 10 or more medicines—so-called hyperpolypharmacy—more than tripled between 1995 (4.9%) and 2010 (17.2%). Polypharmacy is driven largely by multimorbidity, and the associated use of multiple medicines is common in the older population. In addition to increasing the risk of unwanted drug interactions, a wide range of prescriptions can increase a risk of nutritional inadequacy [134]. For example, acid-suppressing drugs and proton pump inhibitors interfere with the status of vitamin B-12, vitamin C, iron, calcium, magnesium and zinc. Anti-hypertensives, including diuretics, decrease the absorption of calcium, magnesium, thiamin, potassium and folate. Anti-diabetic medications, including hypoglycemics and biguanides, interfere with the metabolism of vitamin B-12, calcium and vitamin D. Polypharmacy is also a risk factor for frailty, dry eye and dry mouth [135]. Based on their review, the authors suggest that “In conjunction with an overall healthy diet, appropriate dietary supplementation may be a practical and efficacious way to maintain or improve micronutrient status in patients at risk of deficiencies, such as those taking medications known to compromise nutritional status” [134]. Prevalence of polypharmacy varies among elderly population in Asia from 14.5 % to 86.4%. Further investigations will be required to define any potential problems and to then produce nutrient-based relevant recommended interventions [136,137,138,139].

Oral frailty precedes malnutrition and physical frailty. This decrease in oral function, partially caused by oral diseases such as periodontal disease and candida infection, can lead to difficulty in swallowing (dysphagia), which, in turn, affects food intake, leading to under-nutrition. Subsequent malnutrition then exacerbates the oral health problems, creating a vicious cycle. Micronutrients can play a role in maintaining the different aspects of oral health, including gum health (vitamins A, D and C), dentine (vitamins D and C) and mucosa (vitamins A, B-2, B-3, B-6 and B-12) [140,141,142,143]. The prevalence of periodontal disease increases with age. A Center for Disease Control (US) report from 2012 [144] noted that, in the US, 47.2% of adults aged 30 years and older have some form of periodontal disease and this rate increases to 70.1% for adults 65 years and older. Higher levels of periodontal disease have been reported in Asia and in Oceania [145]. The link between NCDs and periodontal disease has been the subject of a systematic review and it was noted that the rates of dental diseases and NCD prevalence in the various regions of Asia-Pacific varied. In addition, for most of the NCDs, there was a linear significant relationship with periodontal diseases with high statistical significance [146].

Depression has been suggested to contribute to malnutrition through altered appetite related hormone levels that leads to suppressed appetite [147,148]. The proportion of depression in DALY is greater in younger adults (Figure 2). Depression-influenced malnutrition during younger ages could affect health at a later stage, for example, mineral and vitamin D intake and bone mineral density. A meta-analysis indicated that omega-3 LC-PUFA supplementation, especially with EPA, could be beneficial for patients with major depressive disorders, in particular those taking antidepressants [149]. An inverse association has also been reported between dietary omega-3 LC-PUFA intake and prevalence rates of mood disorders in the general population and in perimenopausal women [150,151].

## 7. Potential Approaches to Optimize Nutrient Intake

Because the causes of malnutrition are multifactorial, cross-disciplinary collaboration is required to promote foods with a higher content of micronutrients (quality) and fewer foods purely high in energy (quantity). Intervention strategies are needed to increase access to nutritious foods and micronutrients to ensure dietary adequacy for most individuals in the population and particularly in the older population. Several approaches have been proposed in order to positively influence the nutrient intake behavior. Those include RDA/education, fortification, food labeling and personalized nutritional advice.

National dietary guidelines, such as the Malaysian Dietary Guidelines for the elderly [152] emphasize lower energy intake and providing nutrient-dense foods, having snacks between main meals and ensuring sufficient calcium intake. Recently, an updated DRI for Japan added a chapter on nutritional adequacy for the elderly, with particular mention of protein in relation to sarcopenia and frailty as well as comments on cognitive functions. Dietary Guidelines for Indians also recommend the inclusion of micronutrient-rich foods in the diets of elderly people to enable them to be fit and active [153]. Indonesia and Malaysia have set a higher RDA level for vitamin B-12 to be 4.0 µg/day. There have also been various iterations in revisiting the concept of the Food Guide Pyramid for older people to include, for example, the placement of water at the bottom of the pyramid, because many older adults do not drink enough water to stay hydrated, and putting a flag at the top of the pyramid indicating the need for calcium, vitamin D and vitamin B-12 supplements because many older adults do not get enough of these nutrients in a standard diet. A Modified MyPyramid for older adults had illustrated examples of healthful foods in each food group [154]. It is interesting to note that Malaysia has revised the food pyramid by moving the fruits and vegetables layer, now at level 2, to the bottom of the pyramid. This is to highlight importance of consuming more of these foods for their vitamins and minerals and phytonutrient contents.

Another approach is a nutrient density score label. It aims to help consumers select nutrient-rich foods and promotes healthier diets by enumerating nutritional profile using a scientifically validated tool such as the Nutrient-rich Food Index (NRF 9.3), which is based on nine nutrients to be encouraged and three nutrients to be limited [155]. Front of the pack nutrient labelling has been introduced in several countries including in Australia, despite some caveats in selecting an appropriate index, such as: the applicability of a wide range of food groups, meals and total diets; the fit with a uniform benchmark for mandatory or self-regulation by industry; and applicability as a ‘nutritional navigation system’ for food producers and consumers, helping to optimize products and diets.

Personalized (precision) nutrition has been emerging as a potential tool to drive individual behavioural changes and positively influence an individual’s health. The underlying concept for personalized nutrition is that the link between different phenotypic responses (e.g., weight, blood pressure, etc.) to specific nutritional interventions can be monitored and maintained through measurement of the personal history of markers and behaviours and by adapting nutrition on an individual basis to optimize healthy outcomes [16]. These markers and behaviours include biochemical markers, genetics, dietary habits and eating patterns, circadian rhythms, health statuses, socioeconomic and psychosocial characteristics, food environments, physical activity, and the microbiome. This concept has been adopted by the National Institute of Health in the USA (NIH) as a cornerstone of their direction and funding as a result of a recent report [156]. A set of personal data—for example, blood pressure, biochemical biomarkers, genetic polymorphism and medication intake—can serve as a basis to support a healthy lifestyle. Blood tests have become a basic part of health check-ups in many countries. Nutritional status can be tested as a part of blood biochemical testing and should be part of annual health check-ups. Advances in point-of-care devices and diagnostic tools would enable us to monitor current and potential risks of health. Ten biomarkers have been shown in the algorithm on the basis of their association with chronological age in NHANES III [157]. Where such testing is not feasible, a questionnaire may be utilized as a starting point. Several validated questionnaires are available for screening nutritional status malnutrition in older adults, e.g., MST, MNA-SF and ‘MUST’ [12,15].

The fortification of staple foods is an approach with documented success. In Finland, fortifying milk and dairy products with vitamin D resulted in an increased percentage (91%) of the population reaching the recommended levels of serum vitamin D biomarker concentrations [158]. This action was part of a series of policy-based dietary interventions that were adopted in Finland as a consequence of governmental actions including Food Balance Sheets since 1950s (Ministry of Agriculture and Forestry), FINRISK/Findiet Surveys every 5 years (large population surveys), National Health Behaviour Questionnaires (AVTK)—annual data since 1978 (questionnaire data). The approach has been to work with nutritionists, the population and industries to achieve improved nutrition goals, which was accompanied by extensive public awareness raising and by the active involvement of industries. Similar long-lasting, systematic work, led by the National Nutrition Council and coupled with consumer education for salt reduction demonstrated a decrease by about 30% in Finland since the end of 1970s [159].

Multiple approaches have been proposed in implementing nutritional interventions as a part of health initiatives, and the cost effectiveness of nutritional improvement has been discussed. Since, due to demographic shifts, the potential support ratio is expected to decline, there is increasing recognition of the need for health promotion as a life-cycle approach, and a more prioritised and tailored approach to address the health and well-being of older persons. Such an approach has also been termed “preventive long-term care”. There is, however, still a lack of recognition as to how nutrition may play a role in maintaining healthy parameters related to ageing in existing policies. Several initiatives have also proposed to measure active and healthy ageing with key domains including health, participation, security and environment [4,160,161]. A report sponsored by the United Nations Population Fund (UNFPA) outlined the findings of mapping research that documented the policy, legislation and action plans on ageing and older people in 26 countries across the Asia-Pacific Region [162]. It is noteworthy that there is no specific reference to nutrition and diet in these indices. There is a need to establish clear policies and action plans for the health of the elderly that include nutrition aspects, which will provide guidance to the development for future interventions.

Overall, there is a need for larger numbers of intervention studies among middle and older age groups both to improve existing health and to slow the accumulation of diet/health issues by leveraging the existing data. A successful development of a concept involving early phase intervention with its metrics defined, combined with nutrigenetics and nutritional status check-ups, would enable tailored and effective dietary recommendations to be implemented. For that reason, each intervention should be designed around a primary endpoint with measures focused upon health issues. Secondary endpoints should also be considered since they may provide direct optimization data or an indication of the spread of dose effectiveness. The following is a summary of desirable actions and outcomes to deliver insights to further finetune the recommendation on nutritional intake for healthy ageing. These actions and outcomes should not be considered in isolation or in sequence. They form an overall strategy to be adopted where the different parts are complementary and synergistic.

1. Accurate data on nutritional status, including micronutrient status, in population cohorts of ages from 40 years old onwards. This will provide the baseline of nutrient intake data, and, should it be created, will be managed and held by policymakers. However, the information should be freely available both to public and to individuals so they can determine their own nutrient status. It should be used to define optimal and sub-optimal nutrition.
2. The creation of systematic data on the correlation between nutritional status and NCDs development at different stages in life including early biomarkers of current and future optimum health, healthy resilience and nutritional status. The extent of nutritional insufficiency should be determined through interaction between policy makers and the medical profession. The latter will be a key source of information on actual incidences of potentially diet-related NCDs. While accumulating longitudinal data is important, it is vital to leverage the existing information to support evidence-led interventions on a population basis in some areas. These should be driven by the size of the heath problem, the impact of the potential benefit as well as the weight of scientific evidence. By beginning such interventions now, we will be able to advance the knowledge about the impact and effectiveness of nutritional optimization on healthy ageing and finetune the recommendations in providing benefits to individuals, populations and societies.
3. Intervention carried out by industry and government at a population level focusing on the effect of specific nutrition interventions on the occurrence and progression of NCDs. These interventions will be based upon existing knowledge and also contribute to the development of a new knowledge base in parallel to the above. Based on the existing evidence, we can measure post-intervention improvements in nutritional status. Government, in partnership with the private sector and other stakeholders, can work on specific actions including food fortification and nutrition education in order to optimize the approach and its effectiveness.

A number of longitudinal research projects are in progress among Asian populations which will help to indicate further direction on factors relevant to prolonging healthy longevity [163]. A holistic approach with engaged stakeholders will enable us to determine how nutritional status will affect a number of conditions and to define the range of optimal nutrient intakes that should be as part of a healthy lifestyle, while the ongoing studies accumulate further data and enable more refined recommendations that will be nuanced so as to appeal to consumers and to gain their acceptance.

## 8. Conclusions and Outlook

Asia is a rapidly ageing region and is home to some of the most aged nations in the world, with a wide diversity and range of health issues. Solutions to these many and varied issues, such as NCDs, must be found, which may need to be addressed earlier than at a clinically diagnosable stage in order to address these challenges more cost effectively. Existing evidence suggests that micronutrients play a role in maintaining health and preventing or delaying the onset of leading NCDs that affect this region. Hidden hunger, sufficient energy intake with insufficient micronutrient intake, is particularly relevant in Asia where various levels of nutrient insufficiency exist across the region. Inadequate intakes and low serum concentrations of essential nutrients is a risk factor for both NCDs and the severity of CDs. Optimum nutrition will help improve the health of the individual and improve population health with a concomitant reduction in the health-related costs for an ageing population—which is increasing throughout Asia. There are roles for all the stakeholders, and they must work together to provide synergy between the inputs they make and hence enhance the beneficial outcomes.

Based on existing information, the priority areas for immediate intervention to improve quality of life and to promote health ageing are as follows:

- Vitamin A (under supervision) for immune function and eye health. Deficiency remains to be an issue.
- Vitamin D for musculoskeletal health, heart health and immune function. A significant proportion of the population is at risk for inadequate and deficient status. Intake data is missing in national nutritional survey for multiple countries.
- Vitamin E for diabetes and associated cardiovascular complications, immune function and eye health. A significant proportion of the population is at risk for inadequate and deficient status.
- Vitamin C for diabetes, immune function and eye health. A significant proportion of the population is at risk for inadequate and deficient status.
- B vitamins for cognition and immune function. In particular, vitamin B-12 has a high prevalence of deficiency and impaired absorption with age.
- Zinc for immune function and eye health. Deficiency remains to be an issue. Intake data is missing in national nutritional survey for multiple countries.
- Omega-3 LC-PUFAs for cognition, eye health, heart health and immune function. A significant proportion of the population is at risk for inadequate and deficient status. Intake data is missing in national nutritional survey for multiple countries.

The optimal nutritional status for these nutrients and their synergistic interactions are yet to be unequivocally elucidated. It is important to meet at least the RDI, using supplementation when needed, for each of these micronutrients in order to have an impact upon a number of NCDs. Nutritional advice for healthy ageing should go beyond macronutrients and the limited groups of micronutrients currently being studied and beyond the prevention of nutrient deficiencies.

## Figures and Tables

**Table 1:** Life expectancy and birth rates in selected countries.

Country	Life Expectancy at Birth (2018)	Birth Rate (Births Per Woman, 2018—World Bank)
China	77	1.6
Japan	84	1.4
Singapore	83	1.1
Thailand	77	1.5
Australia	83	1.7
United States	79	1.7
France	83	1.9

**Table 2:** Micronutrient deficiencies and their occurrence in Asia.

Deficiency	Disease	Occurrence
Vitamin A	Night blindness and xerophthalmia	Up to 30% in children in Asia
Vitamin B1	Beri beri. Neurodevelopmental disorders	Up to 50% in parts of East Asia due to diet
Vitamin B3	Pellagra	Comparatively rare but difficult to diagnose in isolation from B complex vitamin deficiency
Vitamin B12	Anaemia	Up to 70% reported in adults in India
Vitamin C	Scurvy	74% of adults in North India and 46% of adults in South India had low vitamin C intake.
Vitamin D	Ricketts	More common in Northern Europe in those of Asian descent.
Calcium	Ricketts and osteoporosis	Low intake in some parts of Asia, e.g., Japan
Iodine	Goitre	Prevalence of 2% in adults and up to 30% among children in some parts of Asia
Iron	Anaemia	Up to 50% among women of reproductive age in parts of Asia
Zinc	Stunting, impaired immunity	Over 25% of inadequate zinc intake in Southeast and South Asian countries

**Table 3:** The availability of micronutrient intake data from national surveys.

	Vitamin A	Vitamin C	Vitamin D	Vitamin E	Vitamin B_1_	Vitamin B_2_	Niacin	Vitamin B_6_	Folate	Vitamin B_12_	Calcium	Iron	Zinc	Omega-3
Australia														
India	*	*			*	*	*		*	*	*	*		
Indonesia	*	*		*	*		*		*	*	*	*	*	
Japan														
South Korea														
Malaysia														
New Zealand	*	*		*	*	*	*	*		*	*	*	*	
Philippines														
Thailand	**	**		**	**	**	**			**	**	**		

Nutrients with intake data available are colored in green. Missing data are represented as grey. * data reported for male and female separately only, ** data reported for both gender combined only.
